# The NtBAG5-CaM complex integrates Ca^2+^ signals to regulate leaf senescence via the antioxidant system in tobacco

**DOI:** 10.1080/21645698.2025.2578048

**Published:** 2025-10-24

**Authors:** Langlang Zhang, Bing Hou, Xiao Chen, Xinghui Liu, Wenxin Xie, Xinxiang Chen, Mingli Chen, Minmin Xie, Jili Zhang, Daping Gong, Quan Sun

**Affiliations:** a Chongqing Key Laboratory of Big Data for Bio Intelligence, School of Life Health Information Science and Engineering, Chongqing University of Posts and Telecommunications, Chongqing, China; b Tobacco Research Institute, Chinese Academy of Agricultural Sciences, Qingdao, China; c China Tobacco Guangxi Industry Co., Ltd., Nanning, China

**Keywords:** Ca^2+^ signals, NtBAG5, NtCaM, senescence, tobacco, yellowing

## Abstract

Leaf yellowing critically impacts tobacco quality and economic value. The Bcl-2-associated athanogene (BAG) gene family regulates plant development and senescence, yet the role of NtBAG5 in tobacco remains poorly understood. Here, we demonstrate that NtBAG5 acts as a key promoter of leaf senescence. CRISPR/Cas9-generated *NtBAG5* mutants exhibited delayed senescence, enhanced activities of antioxidant enzymes, and reduced malondialdehyde (MDA) content, whereas *NtBAG5*-overexpressing plants showed the opposite effects. Promoter-GUS analysis revealed high *NtBAG5* expression in roots and stems. Hormone treatments indicated that *NtBAG5* expression is upregulated by ABA, ETH, IAA, and GA (at late stage) but downregulated by MeJA. Mechanistically, NtBAG5 interacts with calmodulin (CaM) via its BAG domain and IQ motif, as confirmed by yeast two-hybrid and BiFC assays. Ca^2 +^ was found to modulate CaM conformation and strengthen the NtBAG5–CaM interaction. Silencing *NtCaM* via VIGS induced severe leaf yellowing and growth defects. Our results reveal that the NtBAG5-CaM complex, regulated by Ca^2 +^ and hormones, modulates leaf senescence through the antioxidant system, providing new insights for improving tobacco quality.

## Introduction

Plant senescence, the terminal stage of development, is a genetically programmed process essential for nutrient recycling and ecological adaptation.^1,2^ It is characterized by a cascade of irreversible events, including the dismantling of photosynthetic apparatus, degradation of macromolecules, and a well-orchestrated transcriptional reprogramming.^3–5^ This process is intricately regulated by a complex network of internal and external cues. Phytohormones such as ethylene (ETH)^6–8^ and abscisic acid (ABA)^9–11^ act as potent promoters, while cytokinins (CK) and auxins function as repressors.^6,12,13^ Concurrently, reactive oxygen species (ROS) have emerged as pivotal signaling molecules that modulate the senescence program, integrating responses to various environmental stresses.^14–16^

The molecular execution of senescence hinges on the coordinated activation of senescence-associated genes (SAGs) by specific transcription factors. Numerous regulators, including WRKY,^17–19^ NAC,^20–22^ and MYC,^23,24^ have been identified as key nodes that integrate hormonal and ROS signals to direct the senescence process.^4,7,9,10^ Parallel to transcriptional control, protein homeostasis systems, such as the ubiquitin-proteasome and autophagy pathways, also play decisive roles in controlling cellular demise.^25–28^

Within this regulatory landscape, chaperone-mediated protein homeostasis represents a critical layer of control. The BAG (Bcl-2-associated athanogene) family proteins, evolutionarily conserved cochaperones that modulate Hsp70 ATPase activity, are emerging as central regulators of programmed cell death (PCD) and stress responses across kingdoms.^29–31^ Plant BAG proteins have evolved unique structural features and functional diversification.^32,33^ For instance, the Arabidopsis BAG family is divided into two subgroups, with members AtBAG5-7 containing a plant-specific calmodulin (CaM)-binding motif in addition to the BAG domain.^32–35^ Among these, AtBAG5, localized to mitochondria, has been implicated in regulating leaf senescence in an ROS-dependent manner. Its interaction with CaM and Hsc70, sensitive to calcium flux, suggests a mechanism whereby calcium signaling could influence PCD outcomes by altering chaperone activity and ROS accumulation.^36,37^

While the role of AtBAG5 in the model plant Arabidopsis has been preliminarily explored, the function and regulatory mechanisms of its orthologs in crop species remain largely unknown. Tobacco (*Nicotiana tabacum* L.) serves as both a valuable cash crop and a model system for studying leaf senescence and secondary metabolism.^38,39^ Our research group previously conducted a systematic identification of the BAG family in tobacco and discovered that the expression of NtBAG5, the putative ortholog of AtBAG5, is significantly upregulated during leaf senescence. Functional studies revealed that silencing *NtBAG5* enhanced superoxide dismutase (SOD) activity, promoted hydrogen peroxide (H₂O₂) accumulation, and concurrently downregulated the expression of key senescence-markers such as NtSAG12^40,41^ and NtCP1,^42^ ultimately delaying the leaf yellowing process. These findings unequivocally establish NtBAG5 as a positive regulator of senescence in tobacco, potentially through modulating ROS homeostasis and downstream SAG expression.^43,44^

Despite these important advances, the comprehensive molecular network governed by NtBAG5 remains elusive. The upstream signals that regulate its expression and the downstream transcriptional cascades it orchestrates are yet to be defined. To bridge this knowledge gap, this study aims to dissect the precise molecular mechanism by which NtBAG5 regulates leaf senescence. Our work will provide novel insights into the conserved and unique roles of BAG proteins in crop senescence and offer potential strategies for manipulating senescence to improve agricultural traits.

## Materials and Methods

### Plant Materials and Growth Conditions

Tobacco (*N. tabacum*) cultivars K326 and HonghuaDajinyuan, *N. benthamiana* were used in this study. Tobacco seedings were grown on Murashige and Skoog (MS) medium supplemented with 1%(w/v) Suc and 0.8% (w/v) agar, pH 5.8, with 16-h-light (100 μmol m^−2^ s^−1^)/8-h-dark conditions at 25°C. The relative humidity was 60% to 70%. Ten-day seedings were planted in soil/vermiculite/perlite (3:1:1 by volume) substrate for further study.

### NtBAG5 Gene Editing by CRISPR/Cas9

For CRISPR/Cas9-mediated editing of *NtBAG5*, target sequences were cloned into the sgRNA expression vectors pYLsgRNA-AtU6-1 and pYLsgRNA-AtU3b. The purified PCR products containing the target sites were then ligated into the pYLCRISPR/Cas9 vector. The resulting constructs were introduced into *Agrobacterium tumefaciens* strain GV3101 and transformed into tobacco plants K326 via the leaf disc method. Transformed plants were regenerated through tissue culture, and genomic DNA was extracted for amplification and analysis of the *NtBAG5* target region. The same procedure was applied to the *NtPDS* gene as a positive control.

### Overexpression of NtBAG5 in Tobacco

The open reading frame of *NtBAG5* was cloned into tobacco the vector pCAMBIA1300-35S and then transformed into *A. tumefaciens* GV3101. Transformation of tobacco plants (Honghuadajinyuan) was performed using the leaf disc transformation method. The transformed plant materials were cultured to maturity for seed collection. After being subjected to surface sterilization, the harvested seeds were sown on Murashige and Skoog (MS) medium containing kanamycin for selection.

### Seed Germination Experiment

Seeds of Cas9-NtBAG5, NtBAG5-OE, and the corresponding wild-type (WT) tobacco were surface-sterilized by treatment with 70% ethanol for 1–2 minutes, followed by a 10–15 minute soak in 2% sodium hypochlorite (NaClO) solution. After sterilization, the seeds were sown on 1/2 MS medium (supplemented with 1% sucrose and 0.8% agar, pH 5.8) and incubated in a growth chamber at 25°C under a 16 h light/8 h dark cycle. Root length was measured over a period of 14 days. Three biological replicates were set for each line, with 30 seeds per replicate. Statistical analysis was performed using one-way ANOVA followed by Tukey’s multiple comparison test, with significant differences defined as *p* < .05.

### GUS Staining

The upstream promoter region of *NtBAG5* was amplified and cloned into the pCAMBIA1391-GUS vector to generate a promoter-GUS fusion construct. The recombinant plasmid was introduced into *Agrobacterium tumefaciens* strain GV3101 via heat shock and used to transform tobacco through the leaf disc method. Transgenic seedlings were subjected to GUS histochemical staining using a commercial kit (GUS Staining Kit, Solarbio, Cat. No. G3060) according to the manufacturer’s instructions. Stained samples were preserved in ethanol, and the tissue-specific expression pattern of *NtBAG5* was observed directly.

### Phytohormone Treatment

K326 seedlings at the four-leaf stage were sprayed with 10 mg/L solutions of abscisic acid (ABA), indole-3-acetic acid (IAA), gibberellin (GA), methyl jasmonate (MeJA), or ethylene (ETH). Control plants were treated with distilled water under identical conditions. Leaf samples were collected at 0, 1, 6, 24, and 48 hours after treatment. All collected tissues were immediately frozen in liquid nitrogen and stored at −80°C for further use. All experiments were performed with three biological replicates.

### Determination of Physiological Indices in Transgenic Plants

Superoxide dismutase (SOD), catalase (CAT), and ascorbate peroxidase (APX) activities, along with malondialdehyde (MDA) content, were determined using commercial kits: SOD kit (No. A001-1), CAT kit (No. A007-1), APX kit (No. A123-1), and MDA kit (No. A003-1), all purchased from Nanjing Jiancheng Biological Engineering Institute (Nanjing, China). Measurements were performed on 8-week-old plants (*NtBAG5* overexpression lines, knockout lines, and wild-type controls) with three biological replicates per line.

### Virus-Induced Gene Silencing (VIGS) of CAM in Tobacco

Tobacco rattle virus (TRV)-based VIGS was performed using PTRV1 and PTRV2 vectors. A fragment of the CAM gene was cloned into PTRV2 to construct PTRV2-CAM, with empty PTRV2 serving as a negative control. Recombinant PTRV1 and PTRV2 (or PTRV2-CAM) plasmids were co-transformed into *Agrobacterium tumefaciens* GV3101. Bacterial cultures (OD₆₀₀ = 0.8) harboring PTRV1 and PTRV2 derivatives were mixed at a 1:1 ratio, then infiltrated into the abaxial side of 4-week-old tobacco leaves using a syringe. Plants were grown at 22°C under a 16 h light/8 h dark cycle. CAM silencing efficiency was verified by qRT-PCR at 3 weeks post-infiltration.

### Y2H Assay

Protein interactions between NtBAG5 (including domain-truncated variants) and CAM were analyzed using the GAL4 system with pGBKT7 (bait) and pGADT7 (prey; Clontech). Full-length *NtBAG5*, its truncated domains, and full-length CAM were cloned into pGBKT7 and pGADT7, respectively, with all constructs verified by sequencing. Recombinant plasmids were co-transformed into *Saccharomyces cerevisiae* Y2HGold via heat shock. Co-transformants were selected on SD/-Leu/-Trp (double dropout) medium, and positive interactions confirmed by growth on SD/-Leu/-Trp/-His/-Ade (quadruple dropout) medium after 3 days at 30°C.BD-53 + AD-T served as positive control, and BD-lam + AD-T as negative control.

### BiFC Assay

Coding sequences of target genes were cloned into pXY-104-YFP-C (YFP C-terminal fragment) and pXY-106-YFP-N (YFP N-terminal fragment) vectors, respectively, with constructs verified by sequencing. Recombinant plasmids were co-infiltrated into tobacco leaf epidermal cells via *Agrobacterium tumefaciens* (strain GV3101). YFP fluorescence was observed using a fluorescence microscope 48–72 h post-infiltration (25°C incubation). Negative controls included co-infiltration of empty pXY-104-YFP-C and pXY-106-YFP-N.

### Prokaryotic Expression, Purification, and Electrophoretic Mobility Shift Assay (EMSA)

The target gene coding sequence was cloned into pET28a (+), and recombinant plasmids were sequence-verified. Plasmids were transformed into E. coli BL21 (DE3) competent cells. Positive transformants were cultured in LB with 50 μg/mL kanamycin at 37°C to OD₆₀₀ 0.6–0.8, induced with 0.5 mM IPTG, and incubated at 37°C for 4 h. Expressed proteins were analyzed by SDS-PAGE, then purified using the Beyotime His-tag Protein Purification Kit (No. P2229S, Shanghai, China). Purified proteins were supplemented with 10 mM CaCl₂, EGTA, MgCl₂, respectively, followed by PAGE separation, staining, destaining, and visualization.

### RNA Extraction and RT-qPCR

Total RNA was extracted from the leaves of tobacco plants using the FastPure Universal Plant Total RNA Isolation Kit (Vazyme, China) according to the manufacturer’s instructions. First-strand cDNA was synthesized via a two-step protocol with 1 μg of RNA: 4× gDNA wiper Mix treatment (42°C, 2 min) to remove genomic DNA contamination from RNA, followed by reverse transcription using 5× HiScript III qRT SuperMix (37°C, 15 min; 85°C, 5 s) (VAzyme, China). The primers were designed using Primer-BLAST on the NCBI database (https://www.ncbi.nlm.nih.gov/tools/primer-blast/). Relative expression levels were calculated using the 2 ^−ΔΔCt^ method. All primers used in this study were listed in Supplementary Table S1.

### Statistical Analysis

Statistical analysis and data visualization were performed in software GraphPad Prism (v. 8.0.2) (https://www.graphpad.com/) and image J (v. 1.54k). All experiments were performed at least in triplicate. Data are presented as means ± standard deviation (SD). Student’s *t*-test was used to identify significant differences: *p < .05; **p < .01, ***p < .001, ****p < .0001.

## Results

### NtBAG5 Gene Editing and Its Overexpression Promote Leaf Senescence

**Figure 1.**
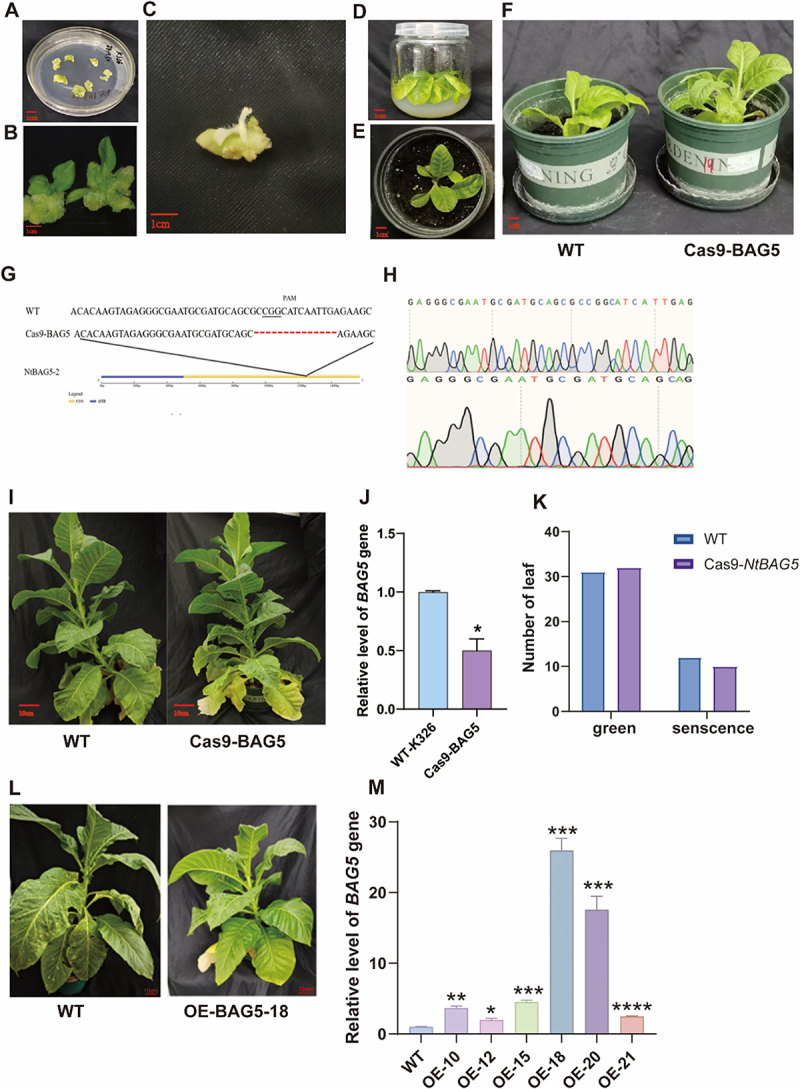
Functional analysis of *NtBAG5* in leaf senescence through gene editing and overexpression (OE). (A, B) Callus formation from leaf discs after *Agrobacterium*-mediated transformation. (C) Albino phenotype of the Cas9-NtPDS positive control seedlings confirming successful CRISPR/Cas9 editing. (D) Rooting Cas9-NtBAG5 resistant seedling. (E) Soil transplantation of Cas9-NtBAG5 resistant seedling. (F) WT and Cas9-NtBAG5 resistant seedling. (G) The target schematic of the *NtBAG5* and sequencing of editing. (H) The schematic of the *NtBAG5* target sequence result. (I) Four months age tobacco seedlings phenotypes of WT and Cas9-NtBAG5. (J) The relative expression level of *NtBAG5* gene in WT and Cas9-NtBAG5 seedling. (K) The number of green and senescent leaves in WT and Cas9-NtBAG5 plants. (L) Four months age tobacco seedlings phenotypes of WT and OE-NtBAG5. (M) The relative expression of *NtBAG5* gene in WT and OE-NtBAG5 lines. Data are presented as mean ± SD from three independent biological replicates. Significant differences compared to WT are indicated as follows: *p* < .05, *p < .01, **p < .001 (Student’s *t*-test).

To further elucidate the role of *NtBAG5* in regulating leaf senescence, we performed leaf disc transformation and observed normal callus formation (Figure 1A,B). Using CRISPR/Cas9-mediated gene editing, with Cas9-NtPDS as a positive control, we obtained albino edited seedlings, confirming the effectiveness of the editing system (Figure 1C). The Cas9-NtBAG5 edited lines were subsequently rooted and transplanted into soil, yielding resistant plantlets (Figure 1D-F). Sequencing of the target sites in these resistant plants revealed a 14-bp deletion at the target locus (Figure 1G,H), indicating successful mutagenesis of *NtBAG5*.

No obvious morphological differences were observed between the *NtBAG5* mutants and wild-type (WT) plants during growth, and both displayed comparable leaf numbers. However, quantitative analysis showed significantly reduced *NtBAG5* expression in the mutant lines. Notably, while lower leaves of WT plants rapidly turned yellow and desiccated, those of the *NtBAG5* mutants exhibited delayed yellowing and slower desiccation, resulting in fewer senescent leaves compared to WT (Figure 1I-K). These results suggest that mutation of *NtBAG5* may retard leaf senescence.

In contrast, overexpression of *NtBAG5* led to premature yellowing of lower leaves. Among the six transgenic lines obtained (lines 10, 12, 15, 18, 20, and 21), significantly higher *NtBAG5* expression levels were detected compared to WT (Figure 1L,M). These findings imply that overexpression of *NtBAG5* may promote early leaf senescence in tobacco.

### NtBAG5 Influences Tobacco Root Growth

**Figure 2.**
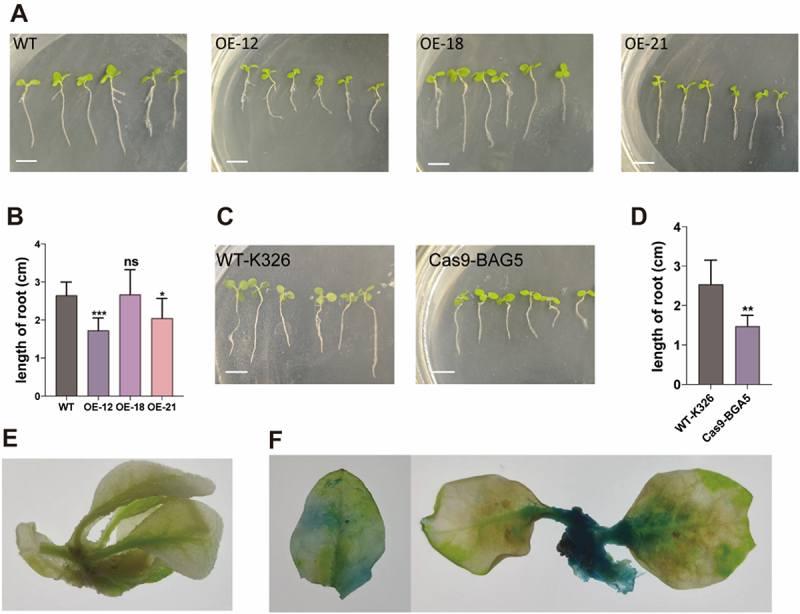
NtBAG regulates tobacco root growth. (A) Representative images of roots phenotypes of WT (honghuadajinyuan), OE-12, OE-18, and OE-21 lines. (B) quantitative analysis of root length of WT (honghuadajinyuan), OE-12, OE-18, and OE-21 lines. (C) Representative images of roots phenotypes of WT(K326) and Cas9-BAG5. (D) quantitative analysis of root length of WT(K326) and Cas9-BAG5. Images were taken 10 days after growth on plates. Scale bars, 5 cm. Asterisks indicate significant differences (*p < .05, **p < .01, ***p < .001, ns: not significant) based on Student’s *t*-test. (E) the GUS staining results of wild-type plants. (F) the GUS staining results of the *NtBAG5* promoter.

Furthermore, during seed germination, we observed that NtBAG5 may play a regulatory role in root growth of seedlings. In overexpression lines, those with up to five-fold increase in *NtBAG5* expression (OE12, OE21) exhibited a significant reduction in root length. Interestingly, when *NtBAG5* expression was substantially higher (over 20-fold compared to the wild type), no significant difference in root length was observed relative to the wild type (Figures 1M, 2A,B). Conversely, gene-edited mutants also showed significantly shorter roots compared to the wild type (Figure 2C,D). These results suggest that NtBAG5 is involved in regulating root growth, but its effect may follow a dose-dependent pattern. The expression pattern of *NtBAG5* was further confirmed by b-glucuronidase (GUS) expression analysis in transgenic tobacco plants. The 1.5-kb upstream fragment of *NtBAG5* was cloned and expressed in the GUS cDNA under the control of this fragment in the plant expression vector. In transgenic tobacco, expression of the *NtBAG5* pro:GUS fusion was detected in the roots, stem, and leaves (Figure 2E,F). Notably, the staining was most pronounced in the root and stem tissues, providing further evidence that NtBAG5 is likely implicated in the process of root development.

### Effects of Hormones on the Expression Level of NtBAG5

**Figure 3.**
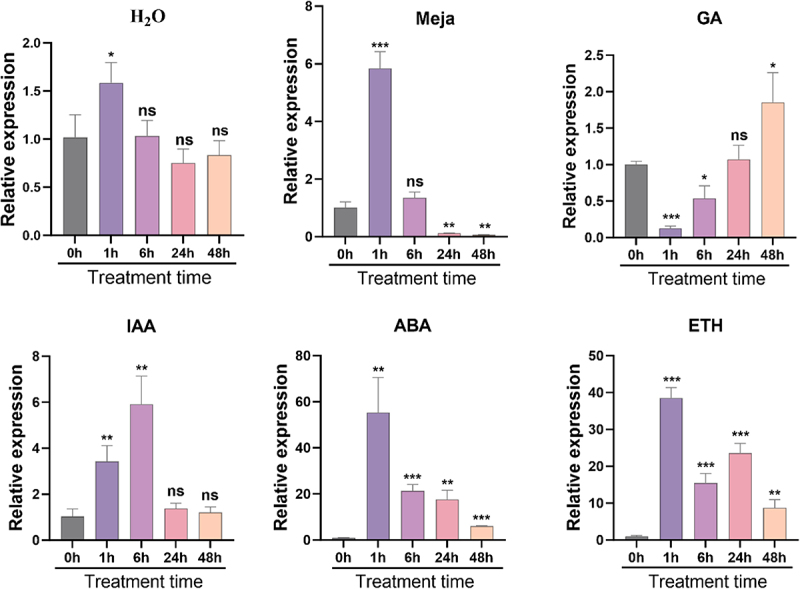
Expression of *NtBAG5* after hormone treatment. (A) Expression of *NtBAG5* after H_2_O treatment for 0–48 h; (B) Expression of *NtBAG5* after MeJA treatment for 0–48 h; (C) Expression of *NtBAG5* after GA treatment for 0–48 h; (D) Expression of *NtBAG5* after IAA treatment for 0–48 h; (E) Expression of *NtBAG5* after ABA treatment for 0–48 h; (F) Expression of *NtBAG5* after ETH treatment for 0–48 h. All data are presented as the mean (± SD) of three independent biological determinations and were analyzed by Student’s *t*-test (*p < .05; **p < .01 and ***p < .001, Student’s t-test.).

**Figure 4.**
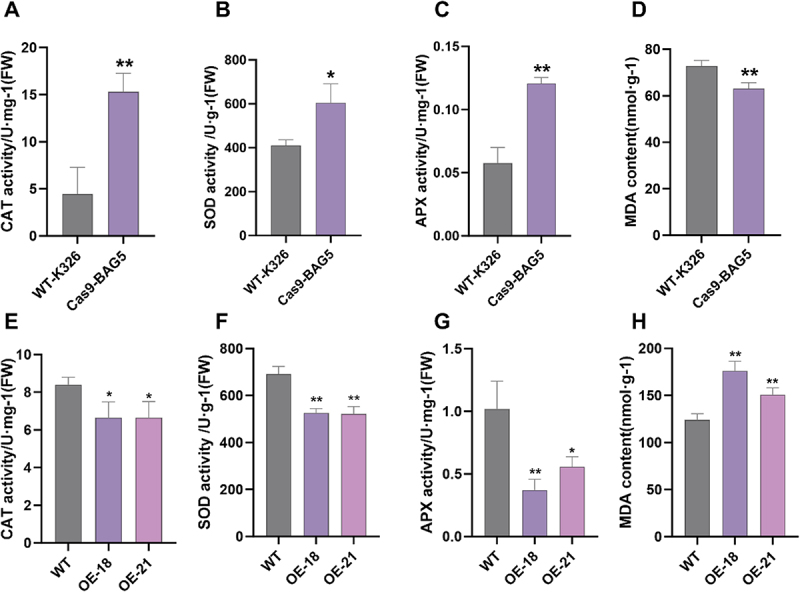
*NtBAG5* regulates ROS and senescence. (A) SOD activity in wild-type tobacco and *Cas9-BAG5* mutant tobacco; (B) CAT activity in wild-type tobacco and *Cas9-BAG5* mutant tobacco; (C)APX activity in wild-type tobacco and *Cas9-BAG5* mutant tobacco; (D) MDA content in wild-type tobacco and *Cas9-BAG5* mutant tobacco; (E) SOD activity in wild-type tobacco and overexpressing *NtBAG5* tobacco; (F) CAT activity in wild-type tobacco and overexpressing *NtBAG5* tobacco; (G)APX activity in wild-type tobacco and overexpressing *NtBAG5* tobacco; (H) MDA content in wild-type tobacco and overexpressing *NtBAG5* tobacco; data are means ± SD from three replicates in (A) to (H). *p < .05; **p < .01 and ***p < .001, Student’s t-test.

Leaf senescence is often closely associated with phytohormone levels. To further investigate the response of *NtBAG5* to various hormones, we examined its expression under different hormonal treatments. In the control group treated with sterile water, *NtBAG5* expression was noticeably upregulated at 1-hour post-treatment, but no significant effect was observed at later time points (Figure 4A). Treatment with methyl jasmonate (MeJA) resulted in a significant downregulation of *NtBAG5* expression between 24 and 48 hours, suggesting that MeJA may suppress both *NtBAG5* expression and senescence (Figure 3B).In gibberellin (GA)-treated plants, *NtBAG5* expression decreased significantly within 1–6 hours, returned to baseline levels by 24 hours, and was subsequently elevated again at 48 hours (Figure 3C), indicating that GA may initially inhibit senescence but promote it at later stages. Following indole-3-acetic acid (IAA) treatment, *NtBAG5* expression increased significantly within 1–6 hours, implying that high concentrations of IAA may promote senescence at early phases (Figure 3D). As expected, both abscisic acid (ABA) and ethylene (ETH) treatments markedly upregulated *NtBAG5* expression (Figure 4E,F), consistent with their known roles in promoting leaf senescence.

### Overexpression and Knockout of NtBAG5 Alter Antioxidant Enzyme Activities and Senescence Progression

Typically, the antioxidant capacity of plants is closely associated with leaf senescence. We found that in *NtBAG5* mutants, the activities of CAT, SOD, and APX were significantly higher than those in the wild type (Figure 4A-C), while the MDA content was markedly lower than in the wild type (Figure 4D). Conversely, in *NtBAG5*-overexpressing plants, the activities of CAT, SOD, and APX were significantly reduced compared to the wild type (Figure 4E-G), and the MDA content was notably higher (Figure 4H). These results suggest that mutation of *NtBAG5* enhances resistance to senescence and oxidative stress, thereby delaying aging. In contrast, overexpression of *NtBAG5* significantly compromises the plant’s oxidative defense system and accelerates the senescence process.

### NtBAG5 Interacts with CaM via Both BAG Domain and IQ Motif

**Figure 5.**
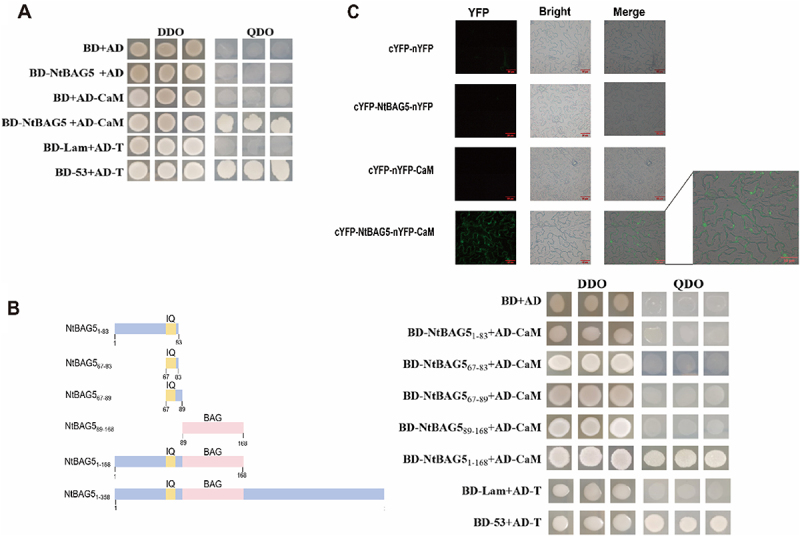
NtBAG5 interacts with CaM via both BAG domain and IQ motif. (A) The interaction of NtBAG5 and CaM in yeast cells; (B) Schematic diagram of NtBAG5 domain truncation and interaction between domain-truncated NtBAG5 and CaM in yeast cells. BD-53+AD-T and BD-Lam+AD-T were used as positive and negative controls, respectively. (C) BiFC analysis of the interaction between NtBAG5 and CaM. Scale bars, 50 μm.

Yeast two-hybrid assays confirmed a specific interaction between NtBAG5 and CaM. All co-transformed yeast strains grew on double dropout medium, but only those containing BD-NtBAG5 + AD-CaM, along with the positive control (BD-53 + AD-T), grew on quadruple dropout medium (Figure 5A). Further domain-truncation analyses revealed that growth on selective medium was only maintained in yeast co-expressing BD-NtBAG5_1−168_ + AD-CaM. No growth was observed with truncations containing only partial regions (BD-NtBAG5_1−83_, BD-NtBAG5_67−83_, BD-NtBAG5_67−89_, or BD-NtBAG5_89−168_) (Figure 5B), indicating that both the BAG domain and the IQ motif are necessary for the interaction.

Bimolecular fluorescence complementation (BiFC) assays in tobacco cells showed strong green fluorescence in samples co-expressing pXY-104-NtBAG5-YFP-C and pXY-106-YFP-N-CaM under excitation light, while no signal was detected in control (Figure 5C). This result further validates the NtBAG5–CaM interaction and suggests that it occurs specifically at the cell wall/cytomembrane/nucleus.

### Potential Involvement of Ca^2+^ in NtBAG5–CaM Interaction

**Figure 6.**
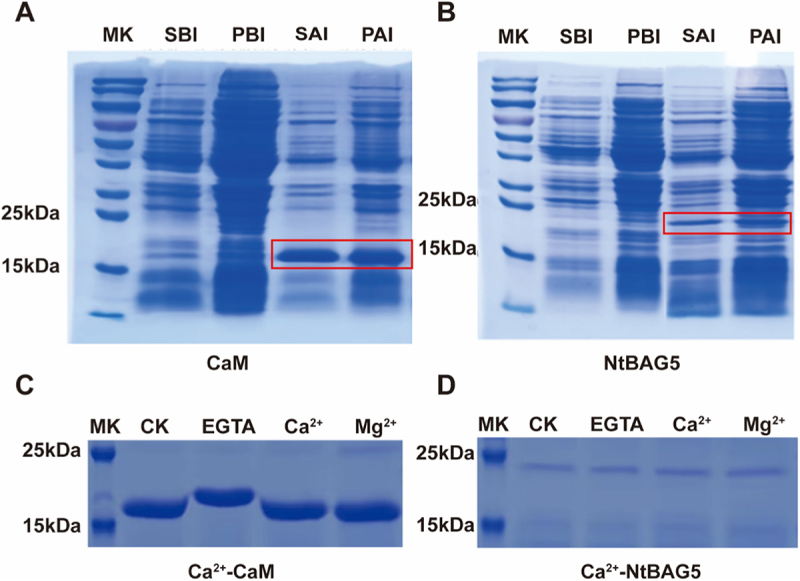
Potential involvement of Ca^2+^ in NtBAG5–CaM interaction. (A) Prokaryotic expression of CaM proteins. (B) Prokaryotic expression of NtBAG5 proteins. Red box showed target protein. (C)The effect of Ca^2+^ on the migration rate of NtBAG5 and CaM proteins. (D) The effect of Ca^2+^ on the migration rate of NtBAG5 and CaM proteins. MK, Marker; SBI, supernatant before induction; PBI, pellet before induction; SAI, supernatant after induction; PAI, pellet after induction.

Given that the NtBAG5–CaM complex can form and that CaM is a typical calcium-binding protein, Ca^2+^ may play an essential role in complex formation and conformational changes. To further investigate the potential binding role of Ca^2+^ in this complex, we obtained recombinant NtBAG5 and CaM proteins through prokaryotic expression, with molecular weights of approximately 18 kDa and 20 kDa, respectively (Figure 6A,B). Electrophoretic mobility shift assays (EMSA) revealed that in the presence of EGTA, the migration of NtCaM was significantly slower compared to the control, Ca^2+^ -supplemented, or Mg^2+^ -supplemented groups (Figure 6C). This suggests that EGTA may further reduce calcium binding to NtCaM, resulting in a more relaxed protein conformation. In contrast, the mobility of NtBAG5 remained unchanged upon EGTA treatment (Figure 6D), indicating that Ca^2+^ does not bind directly to NtBAG5, but rather regulates the complex function through its interaction with NtCaM.

### Silencing of *NtCaM* Promote Leaf Senescence

**Figure 7.**
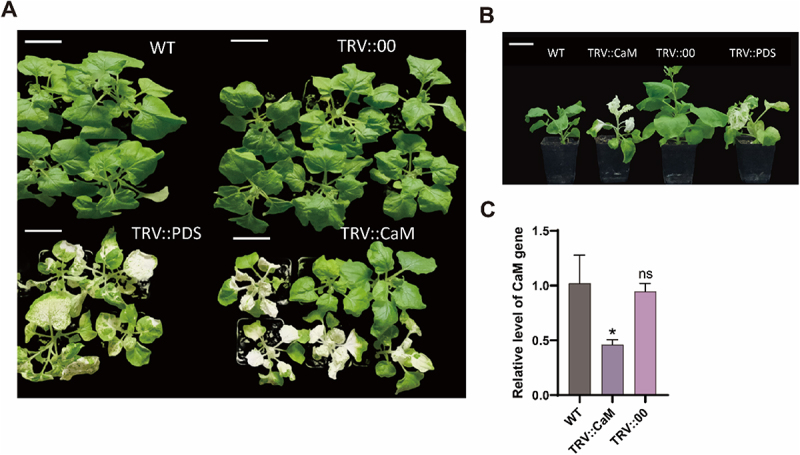
Silencing of *NtCaM* by VIGS can promote leaf senescence. (A) The phenotype of tobacco (*N. benthamiana*) at the seedling stage after silencing of *NtCaM* using VIGS technology. Scale bars, 5 cm. (B) The single seedling phenotype after silencing using VIGS technology. Scale bars, 5 cm. *TRV:PDS* are positive control, *TRV:00* are blank control. (C) Expression of *NtCaM* after VIGS. All data are presented as the mean (± SD) of three independent biological determinations and were analyzed by Student’s *t*-test (*p < .05; ns: not significant).

To determine whether NtCaM within the NtBAG5–CaM complex directly regulates leaf senescence, we employed virus-induced gene silencing (VIGS) to knock down *NtCaM* expression. *NtCaM*-silenced plants exhibited pronounced leaf yellowing, severe growth impairment, and overall reduction in size compared to controls (Figure 7A,B). Although a small subset of plants within the population did not display these symptoms, quantitative analysis confirmed a significant reduction in *NtCaM* transcript levels in silenced individuals (Figure 7C). These results suggest that NtCaM may play a direct role in tobacco leaf senescence.

## Discussion

Leaf senescence in tobacco is a complex process orchestrated by developmental and environmental cues, involving integrated hormone and redox signaling. In this study, we further investigated the functional regulatory network of NtBAG5 and found that it forms a complex with NtCaM. This complex likely undergoes conformational changes upon calcium ion binding and is modulated by hormonal signals, ultimately influencing the activity and levels of antioxidants within the cell and thereby regulating the process of leaf senescence.

### Functional Conservation and Diversification of NtBAG5

BAG proteins are evolutionarily conserved cochaperones regulating stress response and development in plants. In *Arabidopsis*, AtBAG5 localizes to mitochondria and forms a CaM/BAG5/Hsc70 complex that modulates ROS homeostasis and senescence, linking Ca^2+^/CaM signaling to chaperone activity and programmed cell death.^37^ In our previous study, we demonstrated that NtBAG5 enhances ROS accumulation and SOD oxidase activity, forms a complex with heat-shock protein 70 (HSP70), and promotes the upregulation of senescence-associated genes.^43,^ In this current study, we demonstrated that *NtBAG5* is highly expressed in roots and stems, and its overexpression promotes leaf senescence. Investigation of seedling root development revealed that both overexpression and mutation of *NtBAG5* suppress root growth, although extremely high expression levels did not significantly affect root development. These findings suggest that while the function of NtBAG5 is relatively conserved, the tobacco NtBAG5 may have undergone functional diversification.

### Hormone Involved in NtBAG5 Network Regulation Leaf Senescence

Senescence is modulated by multiple hormones: ethylene, ABA, and jasmonate generally promote senescence, whereas gibberellins and auxin often delay it.^45–47^ In this study, we examined changes in *NtBAG5* expression following treatment with MeJA, ABA, ETH, IAA, and GA. The results were largely consistent with expectations. As promoters of senescence, ABA and ETH consistently induced high *NtBAG5* expression, supporting the role of *NtBAG5* in advancing senescence. By contrast, GA was typically considered a senescence-delaying hormone, caused significant downregulation of *NtBAG5* at early stages, which aligns with its anticipated function. Interestingly, IAA treatment triggered a notable upregulation of *NtBAG5* in the early phase. This suggests a dose-dependent effect of IAA, where high concentrations may transiently promote senescence, an observation consistent with previous reports.^47,48^ As Zhang et al. proposed, the regulation of plant senescence is a multi-layered process involving crosstalk between hormone signaling, ROS production, and the expression of senescence-associated genes (SAGs).^49^

### NtBAG5 Promotes Senescence by Suppressing the Antioxidant System

Previous studies using VIGS-mediated knockdown of *NtBAG5* revealed a reduction in ROS accumulation. Since ROS buildup accelerates chlorophyll degradation and activates senescence-associated genes (SAGs), we hypothesized that *NtBAG5* likely influences the antioxidant system. To test this, we examined the activities of key antioxidant enzymes (SOD, CAT, APX) and malondialdehyde (MDA) content in both *NtBAG5*-overexpressing and mutant plants. We found that overexpression suppressed antioxidant enzyme activities and promoted senescence, whereas the *NtBAG5* mutant enhanced antioxidant capacity and delayed senescence. These results indicate that *NtBAG5* acts as an upstream regulator controlling antioxidant capacity and ROS accumulation. This aligns with the established role of AtBAG5 in ROS-related senescence through chaperone complexes, while also highlighting a tobacco-specific connection to CaM/Ca^2+^ signaling.^37^

### The NtBAG5–CaM Complex Regulates Leaf Senescence via Calcium Ion Binding

In Arabidopsis, the AtBAG5–CaM – HSP70 complex promotes ROS production and accelerates leaf senescence under low calcium conditions, whereas high calcium levels induce conformational changes in CaM that suppress senescence.^31,37^ We have previously demonstrated that NtBAG5 can form a complex with HSP70. In this study, we provide evidence that the BAG domain and IQ motif of NtBAG5 interact with NtCaM, and that the conformation of NtCaM changes in response to varying Ca^2+^ concentrations. These findings suggest that NtCaM may also serve as a critical regulator of leaf senescence.^37^ This conclusion is consistent with reports in Arabidopsis indicating that Calmodulin 1 regulates both senescence and ABA response.^50,51^ Furthermore, VIGS-mediated knockdown of *NtCaM* resulted in obviously leaf yellowing in most treated plants. However, CaM belongs to a large protein family involved in diverse processes, including biotic and abiotic stress responses, growth development, and basic metabolism. Our results imply that NtCaM may integrate hormonal and stress signals, activate ROS production, and ultimately promote senescence in response to fluctuations in Ca^2+^ levels.

## Conclusions

In conclusion, our study establishes NtBAG5 as a central positive regulator of leaf senescence in tobacco. We propose a model wherein the NtBAG5–CaM complex, situated at the cell wall, acts as a key signaling node that integrates calcium fluctuations and hormonal signals (e.g., ABA, ETH) to dampen the antioxidant system, leading to ROS accumulation and ultimately triggering senescence. This work not only elucidates a novel Ca^2+^/CaM-dependent senescence pathway in tobacco but also identifies the NtBAG5–CaM module as a potential target for genetic strategies aimed at delaying senescence and improving crop quality. Moreover, these findings provide a foundation for further dissection of the molecular network underlying leaf senescence in plants.

## Supplementary Material

Supplementary Table 1 primers list1.xls
